# Radiation exposure for planning deformity corrections with hexapods: comparison of conventional X-ray, standard CT, and ultra-low-dose CT

**DOI:** 10.1007/s00402-026-06479-x

**Published:** 2026-09-23

**Authors:** Daniel Schüll, Heiko Baumgartner, Belinda Krüger, Julian Thelen, Gabriel Keller

**Affiliations:** 1 Department of Traumatology and Reconstructive Surgery, BG Trauma Center Tübingen, Eberhard-Karls University of Tübingen, Schnarrenberg-Str. 95, 72076 Tübingen, Germany; 2 Departement of Radiology, Universitätsklinikum Tübingen, Tübingen, Germany

**Keywords:** Diagnostic imaging, Long bones, Deformity analysis, Radiation protection, Taylor spatial frame

## Abstract

**Introduction:**

Hexapod systems are among the most accurate devices for correcting complex bone deformities. Postoperative planning is carried out using web-based software solutions based on at least two X-ray images in orthogonal planes. Recent studies have shown that inaccuracies in determining mounting parameters and the spatial relationship between the reference ring and the bone may lead to measurement errors. Computed tomography images potentially offer greater accuracy in this regard, but are associated with increased radiation exposure. The aim of this study was to compare radiation exposure from conventional radiography (X-ray), standard computed tomography (CT), and ultra-low-dose CT (ULD-CT) in the context of planning deformity corrections using a hexapod system and to evaluate suitability and comparability of these imaging modalities for treatment planning.

**Materials and methods:**

Between March 1, 2022, and August 31, 2024, 28 patients were treated with a hexapod system for post-traumatic deformities at a Level I trauma center. All patients for whom standardized radiographs (anteroposterior and mediolateral views), conventional CT scans, and ULD-CT scans were available were included in this retrospective analysis. The study population thus comprised *n* = 18 patients. The effective radiation dose (µSv) was calculated separately for each examination. The values were compared using descriptive statistics and inferential analysis (repeated-measures ANOVA; *p* < 0.05).

**Results:**

The mean effective dose was 1.41 µSv (min 0.66 µSv, max 2.53 µSv, SD ± 0.6 µSv) for conventional X-rays, 4.61 µSv (min 2.97 µSv, max 6.68 µSv, SD ± 1.00 µSv) for ULD-CT, and 150.8 µSv (min 94.85 µSv, max 224.6 µSv, SD ± 35.81 µSv) for standard CT. ULD-CT showed significantly lower radiation exposure than standard CT while offering sufficient image quality for treatment planning. Although the radiation dose of ULD-CT was higher than that of X-rays, but in relation to the measurement precision achieved, there was a clear reduction in dose compared to standard CT.

**Conclusion:**

ULD-CT provides image quality suitable for treatment planning comparable to standard CT with significantly reduced radiation exposure. Given the significantly improved planning quality and acceptable dose, ULD-CT can be recommended as a favorable balance between precision and radiation protection. In conclusion, ULD-CT represents a valuable imaging option for obtaining the most accurate determination of the distance between two points, particularly when defining mounting parameters.

## Introduction

Radiological imaging diagnostics has become an indispensable part of modern medical practice. It supports diagnosis, facilitates treatment and can document treatment progress or changes. As an additional tool, imaging can reduce the risk of overlooking diseases and injuries, thereby reducing diagnostic errors. However, these benefits depend on accurate image acquisition and expert interpretation. In addition, there are downsides to the ever-increasing availability of imaging, both locally and temporally. Adequate clinical assessment is often sacrificed in favor of immediate imaging. More importantly, the growing use of ionizing radiation in diagnostic and therapeutic procedures has substantially increased radiation exposure for both patients and healthcare professionals [1–3]. A large proportion of this exposure is attributable to imaging performed in orthopedic and trauma surgery [4].

However, orthopedics (and trauma surgery in particular) depends on adequate and advanced imaging for diagnostics and therapy planning. Consequently, the judicious use of modern imaging techniques that minimize radiation exposure while maintaining diagnostic quality is essential [5]. In the field of orthopedic and trauma surgery, imaging is a fundamental component of diagnosis and therapy planning. This applies to acute cases as well as chronic, post-traumatic or congenital deformities of the skeleton. In the context of deformity correction, especially of the extremities, imaging plays a pivotal role throughout the treatment process and is therefore a major determinant of both functional and clinical outcomes.

Modern hexapod systems such as the Taylor Spatial Frame (TSF) enable three-dimensional correction of complex deformities through accurate determination of deformity and mounting parameters. They are the most accurate devices for performing limb lengthening or deformity correction [6]. To exploit the full potential of the procedure, deformity parameters, mounting parameters, and the center of rotation and angulation (CORA) must be determined with the highest possible precision relative to the reference ring. The more accurate the measurements are, the more precise the correction plan will be. Since treatment using hexapod systems lasts several months and requires numerous follow-up imaging examinations, radiological diagnostics must meet high standards to deliver the lowest possible radiation dose while maintaining the highest possible image quality, in accordance with the ALARA (As Low As Reasonably Achievable) principle.

The use of inaccurate deformity and mounting parameters can result in imprecise deformity correction, leading to residual or secondary deformities [7, 8]. Ferreira et al. described the current gold standard for deformity analysis using SMART-TSF (Smith & Nephew, Inc. 1450 Brooks Road, Memphis, TN 38116 USA) in combination with the latest version of the SMART-TSF web application and their latest device, the Beacon [2]. Baumgartner et al. demonstrated that a CT scan provides the most accurate measurements of both mounting and deformity parameters [9–11]. The accuracy of these measurements depends largely on the quality of the imaging. Traditionally, planning is based on two orthogonal X-ray images, but geometric distortion and perspective deviations can limit the planning accuracy. Disadvantages of X-rays are image overlap within the region of interest- particularly due to a relatively large hexapod system- or the risk of inaccurate aligning the X-ray beam. This can lead to repeated image acquisition and consequently increasing radiation exposure. Several studies have shown that computed tomography (CT) is superior to conventional X-ray imaging in terms of planning accuracy and overall evaluability. The disadvantage of CT has traditionally been its higher radiation exposure, which which has limited its routine use, especially in pediatric and younger patients [12].

The average radiation dose, consisting mainly of cosmic, terrestrial, radon gas and incorporated radionuclides (e.g., potassium-40 in food), is approximately 2–3 mSv per year in Europe, with regional variations. Artificial, medical, and technical sources add a further dose of around 2 mSv per year, resulting in a total annual exposure of approximately 4 mSv per person [10, 13, 14]. Despite recent advances in dose reduction, it remains unclear whether ultra-low-dose CT provides planning accuracy comparable to standard CT while maintaining radiation exposure at a level comparable to conventional radiography. One innovation is the use of a new low-dose CT protocol, which enables a significantly lower dose while maintaining sufficient image quality through optimized parameters [15]. The aim of this study was to compare the radiation exposure associated with conventional radiography, standard CT, and ULD-CT and to evaluate the agreement and clinical applicability of these methods for deformity correction using hexapod systems.

## Materials and methods

At our facility, we have already incorporated ULD-CT scanning into our protocol for correcting deformities. Kin some cases we had to perform a standard CT scan to determine the progress of calcification in the distraction callus. We also obtained native X-rays for patients whose angulation had been corrected. For this study, we were able to retrospectively include 18 patients who underwent all three imaging methods: X-rays, standard CT scans, and ULD-CT scans of their lower extremities.

### Study population

A total of 28 patients underwent surgical treatment with the Taylor Spatial Frame (TSF) at a Level I trauma center (BG Trauma Center Tübingen) between March 1, 2022, and August 31, 2024. All consecutive patients with clinically indicated post-traumatic deformity corrections of the lower extremities were included. The exclusion criterion was the absence of one or more of the imaging modalities evaluated (conventional X-ray, standard CT scans, ULD-CT scans). The final study group consisted of 18 patients (*n* = 18).

### Technical parameters of the standard protocol for CT of the lower extremity

Standard CT imaging was performed using a 128-slice single-source CT scanner (SOMATOM Definition Edge, Siemens Healthineers, Munich, Germany) using a standard examination protocol with a tube voltage of 120 kV and a tube current of 95 mAs. The pitch was 0.8, the rotation time was 1.0 s, and the collimation was 128 × 0.6 mm. No iterative image reconstruction was used. The slice thickness was 2 mm, a very sharp reconstruction kernel (B75h) was applied, and images were reconstructed.

### Technical parameters of the ULD-CT protocol

A previously developed and evaluated ULD-CT protocol for torsional imaging of the lower extremities was adapted for the present study [16]. ULD-CT were performed using the same 128-slice single-source CT scanner (SOMATOM Definition Edge, Siemens Healthineers, Munich, Germany). Scan parameters included a tube voltage of 80 kV, a pitch of 1.0, a rotation time of 0.5 s, and a detector collimation of 128 × 0.6 mm. Automatic tube current modulation (CARE Dose4D, Siemens Healthineers, Munich, Germany) was used with a reference setting of 20 mAs, which was adjusted to optimize the visualization of bone structures. In addition, raw-data based iterative image reconstruction (SAFIRE – Sinogram Affirmed Iterative Reconstruction, Siemens Healthineers, Munich, Germany)was performed at strength level 3. Images were reconstructed with a slice thickness of 2 mm using a medium-sharp reconstruction kernel (I50f) and displayed in a bone window.

### Technical parameters of the X-ray examinations

X-ray examinations were performed on a digital X-ray unit (Fluorospot Compact, Siemens Healthineers, Munich, Germany) using a standard protocol for the lower leg and knee. Automatic exposure control was used with reference settings for both anterior-posterior (AP) and medial-lateral (ML) view of 60 kV and 4 mAs. The images were taken with the patient lying on the X-ray table using an under-table grid.

### Estimation of effective radiation exposure from X-ray examinations

The dose-area product of the ap view and the lateral views was added together to obtain a total dose-area product. This was multiplied by the region-specific conversion factor k for standard X-rays of the knee in accordance with the British Health Protection Agency Report from 2011 [17], and with reference to Publication 103 of the International Commission on Radiological Protection from 2007 [17, 18]. The tissue-specific weighting factors of the anatomical structures in the examination area, which are decisive for determining the effective dose are incorporated into this calculation.

### Estimation of effective radiation exposure from CT and ULD-CT examinations

In axial reconstructions, the maximum diameter of the lower leg was measured in the anterior-posterior and medial-lateral directions. From these measurements, the effective diameter of the lower leg was calculated as the square root of the product of the two measured values, multiplied by two, as both lower extremities are usually included within the scan field during lower-leg CT examination. The corresponding conversion factor (f) was obtained from AAPM Report 204 [19, 20], using the 32 cm model implemented by the scanner. The Size-Specific Dose Estimates (SSDE) was calculated as the product of the CT dose index volume (CTDIvol) and this conversion factor f. Multiplication of the SSDE by the scan length yielded the adjusted Dose-Length Product (DLP). The DLP was subsequently multiplied by the conversion factor (k) for knee examinations to determine the effective dose of the CT examination [21].

### Evaluation of the comparability of the procedures in terms of planning

The image quality of the ULD-CT was assessed dichotomously (“adequate” vs. “inadequate”) with regard to the accuracy of assessment of cortical bone alignment for treatment planning. The evaluation was conducted in a blinded manner and in random order. The reviewers were the senior orthopedic surgeon responsible for lower-extremity deformity corrections (H.B., 22 years of experience in surgical deformity correction) and a radiologist specialized in musculoskeletal radiology (G.K., 11 years of experience in specialized musculoskeletal imaging). In this regard, all datasets (100%) were considered diagnostically sufficient for treatment planning. The ULD-CT examination protocol has already been implemented in the clinical routine at our clinic, supporting its clinical applicability. It remains to be seen whether treatments based on ULD-CT planning lead to higher complication rates. These clinical outcome data are currently being collected prospectively and will be analyzed in a future study.

### Statistics

The data were analyzed using SPSS 29.0 (IBM, Armonk, NY). The Shapiro-Wilk test was used to assess normal distribution. Comparisons were performed using ANOVA with Bonferroni correction; the significance level was set at *p* < 0.05. In cases where normal distribution was not present, the Friedman test was performed. This applied to the gender variable. The remaining data were normally distributed, so an ANOVA with repeated measures was performed. Variance homogeneity was checked using the Levene test. In cases of variance heterogeneity in the Levene test, the Welch test was performed. In cases of sphericity violation, a Greenhouse-Gaisser correction was applied. The data were presented according to the mean (standard deviation, minimum–maximum) format.

## Results

A total of 18 patients (15 male, 3 female) with an average age of 48.5 years ± 16.2 years (mean: 48.5; range: 22.0–72.8; SD: 16.2)) were included in the analysis.

The mean effective dose for both X-rays (AP and ML projections) was in total 1.41 µSv (min 0.66 µSv, max 2.53 µSv, SD ± 0.6 µSv) with a normal distribution according to the Shapiro–Wilk test (*p* = 0.18). The mean effective dose for standard CT was 150.8 µSv (min 94.85 µSv, max 224.6 µSv, SD ± 35.81 µSv).

The mean effective dose for ULD-CT was 4.61 µSv (min 2.97 µSv, max 6.68 µSv, SD ± 1.00 µSv).

The groups were evaluated using ANOVA with repeated measures for dependent samples and a post hoc test according to Bonferroni. The ANOVA with repeated measures and Greenhouse-Geisser correction showed that the average dose differed significantly. F F(1.002, 17.023) = 302.5, *p* < 0.001, partial η² = 0.95. A Bonferroni-corrected post-hoc test showed a significant (*p* < 0.001) dose reduction between standard CT imaging and X-ray imaging (mean difference: 149.4 µSv, SD: ±8.5 µSv). A significant (*p* < 0.001) dose reduction **of** approximately 30-fold was also demonstrated between standard CT imaging (mean difference: 146.2 µSv, SD: ±8.5 µSv) and ULD-CT. Similarly, the Bonferroni-corrected post-hoc test showed a significantly (*p* < 0.001) higher radiation dose for ULD-CT imaging (mean difference: 3.2 µSv, SD: ±0.3 µSv) compared with conventional X-ray imaging, representing a difference by a factor of 3.

**Fig. 1:**
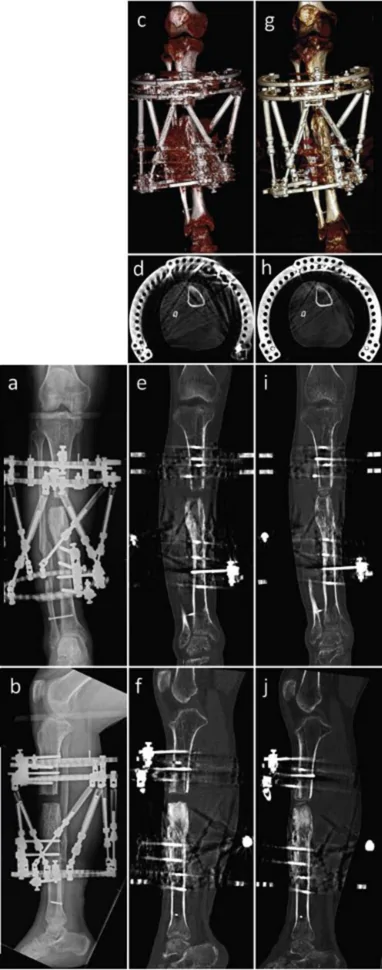
Comparison and illustration of the radiological modalities performed: comparison and illustration of the radiological modalities performed: **a**, **b** conventional X-ray imaging anterior-posterior and medial-lateral; **c**–**f** ULD-CT as 3D model, axial, coronal, and sagittal; **g**–**j** conventional CT as 3D model, axial, coronal, and sagittal

For illustrative purposes, conventional X-ray imaging is shown in Fig. 1 below. Images a and b show conventional X-ray imaging. Images c-f illustrate ULD-CT imaging using a 3D model as well as axial, coronal, and sagittal reconstructions. Images g-j compare conventional CT imaging in the same plane.

All ULD-CT scans were deemed by both the orthopedic surgeon and the radiologist to be “adequate” for assessing cortical alignment within the context of treatment planning.

## Discussion

Shortly after the discovery of X-rays by Wilhelm Conrad Röntgen in 1895 scientists already suspected that they could also be hazardous. As early as 1896, 55 radiation-induced injuries had occurred [22]. The first harmful effects of X-ray exposure were reported only one month after their discovery (16). Injuries ranging from skin burns and hair loss to necrosis, eye irritation and cancer have been published [23, 24]. Since then radiation dose and exposure time has been a major focus of radiation protection strategies. In the early days of radiography exposure times of 30 up to 120 min and radiation doses of up to 15000mSv per single X-ray examination were not uncommon [25–29]. It has been hypothesized that individuals during that period may have received cumulative absorbed doses of up to 3000 rads (30000mSv) over their livetime [30]. Fortunately, we learned a lot in the past 125 years. The transition from film-based to digital imaging, the use of collimation and improvements in radiation protection have reduced the effective dose of a two-view chest x-ray examination to approximately 0,1 mSv, which corresponds to about 10 days of natural background radiation that we are all exposed to as part of our daily living [31]. An x-ray of the lower leg with an effective dose of 2 µSv equals 0,002mSv and a CT-scan of a lower leg with an effective dose of 200 µSv equals 0,2 mSv. The annual natural background radiation exposure is approximately 2 mSv and the annual limit values (effective dose) for occupationally exposed persons in Germany is 20 mSv (according to § 78 Radiation Protection Act). Exposure to multiple CT scans and other sources of low-dose radiation with a cumulative dose up to 100 mSv and possibly up to 200 mSv, has not been shown to increase cancer risk [32]. CT evaluation, with or without 3D reconstruction, is a crucial tool in complex bone reconstruction and deformity treatments. Therefore, strict indications are necessary to reduce radiation exposure [33]. In this context, the challenge remains to balance the diagnostic benefits of CT-based deformity analysis with the need to minimize radiation exposure, particularly in patients requiring repeated imaging during prolonged treatment courses.

This is why we developed this ULD-CT protocol reduce the effective radiation dose by approximately thirtyfold compared with standard CT.

This study shows that ULD-CT has a significantly lower radiation exposure than standard CT, while simultaneously providing image quality that, in the view of an experienced orthopedic surgeon and an experienced radiologist, is sufficiently high for the precise planning of corrective procedures for deformities of the lower extremity in routine clinical practice. The results are consistent with and extend previously published data on dose reduction in musculoskeletal CT diagnostics [8, 11, 12, 15, 19, 21]. While conventional CT in this study caused the highest radiation exposure with an average effective dose of 150.8 µSv, the dose of ULD-CT was approximately 30 times lower and therefore within a range that has rarely been achieved for CT-based planning procedures of the extremities. Nevertheless, the dose of ULD-CT was about three times higher than that of conventional X-rays, which must be interpreted in the context of its significantly higher planning reliability and accuracy. In addition, unlike X-rays, ULD-CT enables the possibility of subsequent multiplanar image reconstruction, if considered necessary by the surgeon. Furthermore, unlike X-rays, it enables three-dimensional reconstruction for the visualization and assessment of complex deformities.

### Comparison with existing evidence on CT dose reduction

The literature increasingly shows that low- and ultra-low-dose CT (ULD-CT) protocols can be used successfully in musculoskeletal diagnostics, both in fracture diagnosis and in deformity analysis. Konda et al. [11] demonstrated as early as 2016 that ULD-CT examinations can detect fractures of the extremities with high diagnostic certainty – at an effective dose that was, in some cases, lower than that of multi-projection radiography. A recent meta-analysis confirms the suitability of low-dose CT protocols for fracture diagnosis, demonstrating a significant reduction in dose [12]. In pediatric imaging, Waelti et al. [22] were also able to show that ULD-CT protocols for torsion measurement deliver results that are just as accurate as standard CT protocols.

The present results support these developments and show that ULD-CT also provides a reliable image basis for patients with complex deformities. ULD-CT offers a significantly more precise data basis than conventional X-rays, particularly for determining mounting parameters and the CORA determination - a result that is consistent with the findings of Baumgartner et al. and Mederake et al. [13], who demonstrated improved measurement accuracy with CT-based assessment compared with conventional radiography.

### Significance for the planning of deformity corrections

The accuracy of deformity analysis is a key success factor in treatment using hexapod systems such as the TSF. The present study demonstrates that, despite low radiation exposure, X-rays have significant limitations in terms of perspective distortion, measurement errors due to rotational inaccuracies, and lack of reproducibility – limitations that have already been described in earlier studies [1–3].

Although standard CT diagnostics is considered the gold standard in terms of measurement accuracy, it has been of limited use for routine planning due to the associated radiation burden. The introduction of ULD-CT may help to close this gap: the 30-fold reduction in dose compared to standard CT shown in this study, without compromising diagnostic assess ability, represents a significant advance. This circumstance becomes even more relevant in the course of therapy, as the cumulative radiation exposure increases with the repeated imaging examinations required throughout the treatment period.

The planning accuracy of ULD-CT was superior to conventional X-ray diagnostics. The usability of ULD-CT diagnostics in terms of planning accuracy was considered adequate by both reviewers (100%). The procedure is already firmly established in our daily clinical practice and workflow and has already been evaluated with regard to planning methods and interpretability for other injuries [34, 35].

Ultimately, it remains to be seen whether a higher revision rate will be observed in the future following deformity corrections using ULD-CT compared to standard CT diagnostics. Further studies are already being planned to evaluate planning reliability.

In our setting, the image quality—which is considered comparable to that of standard CT diagnostics—enables reliable planning options with a lower radiation dose. This is consistent with the current scientific literature, which describes the superior performance of CT-based methods, particularly in the measurement of misalignments and in three-dimensional planning procedures [7, 13, 21].

Another advantage of CT-based methods is that they function independently of external referencing instruments. This avoids projection-related distortions and superimposition artifacts inherent to two-dimensional radiography, reducing the need for repeated image acquisition—an aspect that contributes to dose reduction in practice.

### Radiation exposure in a clinical context

The average natural radiation exposure in Germany is around 2 mSv per year; medical applications contribute a further approximately 2 mSv/year [10, 14, 20]. In comparison, the absolute doses of ULD-CT determined in this study are very low and are in the range of typical routine radiographic diagnostic procedures in orthopedics. At the same time, it should be noted that cumulative radiation exposure may still be relevant in young patients or in the context of repeated follow-up examinations, which is why the ALARA (“As Low As Reasonably Achievable”) principle continues to apply and conventional radiography remains useful for follow-up examinations due to its low radiation dose [4, 9].

### Clinical consequences and future developments

The results of the study suggest that ULD-CT represents an favorable compromise between radiation protection and diagnostic precision for the planning of hexapod-system-based deformity corrections. It is particularly noteworthy that the image quality achieved showed no limitations in terms of evaluability, thus the application of CT-based planning for the first time with substantially reduced radiation exposure in routine clinical practice.

The ULD-CT protocol used in this study can be applied on standard CT-systems and does not require any special (and often expensive) software or hardware components. With the help of such dose-optimization strategies, it is possible to further reduce radiation doses to the level of conventional X-ray diagnostics [15, 19]. In the long term, CT diagnostics could potentially become an important tool not only for primary deformity planning but also for selected follow-up examinations. We achieve this dose reduction without compromising the diagnostic value of the examination and without the need for special hardware or software components. Future developments, including photon-counting CT, spectral filtration, and AI-based reconstruction algorithms, may further reduce radiation exposure while preserving or even improving image quality.

## Conclusion

The results of this study are consistent with the current scientific literature and demonstrate that ULD-CT is an accurate imaging modality that provides substantial radiation dose reduction for planning complex deformity corrections. This technology represents a promising alternative to conventional imaging approaches by combining high diagnostic accuracy with reduced radiation exposure. Looking ahead, ULD-CT protocols may contribute to further dose optimization and expand the role of CT-based imaging in musculoskeletal diagnostics. Long-term clinical outcomes, including potential effects on complication and revision rates, require further evaluation. These data are already being systematically collected and will be evaluated as part of a future study.

## Limitations

The study has several limitations. The most significant limitation is the dichotomous assessment of image quality and suitability for treatment planning. Although the evaluation was conducted by experts from various medical disciplines with many years of professional experience, an objective measurement of image quality is a necessary complement to this study and is already underway as part of further research. Second, the actual clinical impact of the various planning methods (e.g., the accuracy of the final correction) was not assessed. Future prospective studies should therefore systematically analyze clinical outcomes and correction accuracy and investigate the possibility of further dose reduction in ULD-CT through the use of specialized hardware and software components, such as AI-based reconstruction algorithms [34–36]. Third, the retrospective design of this study limits the generalizability of the findings [36–38].

## Data Availability

All data supporting the findings of this study are available within the paper.
